# Elevated levels of procoagulant microparticles in a patient with myocardial infarction, antiphospholipid antibodies and multifocal cardiac thrombosis

**DOI:** 10.1186/1477-9560-3-15

**Published:** 2005-10-11

**Authors:** O Morel, L Jesel, JM Freyssinet, F Toti

**Affiliations:** 1 Fédération de Cardiologie des Hôpitaux Universitaires de Strasbourg, Hôpital de Hautepierre, 1 avenue Molière, 67098 Strasbourg, France; 2 Université Louis Pasteur, Faculté de Médecine, Institut d'Hématologie et d'Immunolgie, 4, rue Kirschleger, 67085 Strasbourg, France; 3 Unité 143 INSERM, Hôpital de Bicêtre, 78, rue du Général Leclerc, 94275 Le Kremlin Bicêtre, France; 4 Université Paris-Sud, Faculté de Médecine, 63 rue Gabriel Péri, 94270 Le Kremlin Bicêtre, France

**Keywords:** endothelium, acute coronary syndrome

## Abstract

Circulating procoagulant microparticles (MP) are pathogenic markers of enhanced coagulability associated to a variety of disorders and released from stimulated vascular cells. When derived from endothelial cells, MP were found characteristic of thrombotic propensity in primary antiphospholipid syndrome (APS). The prothrombotic status of a patient with antiphospholipid antibodies (APL), a past history of mesenteric vein thrombosis and presenting myocardial infarction and extensive intracardiac thrombosis was examined by measurement of circulating procoagulant MP. MP of platelet and endothelial origins were highly elevated with respect to values detectable in patients with myocardial infarction and no history of APS (6- and 3-fold elevation, respectively) or in healthy volunteers (13- and 25-fold elevation, respectively). In this particular patient, with moderate APL titer, a drastic release of procoagulant MP could have contributed to thrombus growth and the development of extensive intracardiac thrombosis.

## Introduction

In primary anti-phospholipid syndrome (APS), arterial or venous thrombosis and recurrent pregnancy loss are common thrombotic manifestations associated with anti-phophospholipid antibodies (APL) [1]. A variety of cardiac affections including valvular lesions, myocardial dysfunction or infarction, early bypass graft failure, and intracardiac thrombosis have been reported [2-4]. Yet *in vitro*, APL inhibit phospholipid-dependent blood coagulation and bind to membrane phospholipids exposed by stimulated or apoptotic cells [5]. Furthermore, in the presence of APL, vascular cells are stimulated and acquire procoagulant, proadhesive and proinflammatory phenotypes [1,6]. Two non-exclusive hypotheses have been proposed to explain the thrombotic propensity in APS (i) APL could impede the natural anticoagulant protein C pathway, also involving anionic phospholipids (ii) APL would promote sustained cell activation through the interaction of immune complexes with vascular cells [1,7].

Procoagulant microparticles (MP) are shed from the plasma membrane of any stimulated or apoptotic cells [8,9]. Under most physiopathological conditions among which acute myocardial syndrome, circulating MP, are mainly of platelet origin [10,11] and can be viewed as a "storage pool" by themselves, disseminating procoagulant activities [9,12]. In blood flow, released MP contribute to thrombotic propensity by virtue of exposed anionic phospholipids enhancing the catalytic surface available for blood coagulation. In addition, harbored membrane glycoproteins or antigens cytoadhesins, and proinflammatory lipids confer to MP the potency of cellular effectors through multiple amplification loops [13].

Elevated levels of circulating MP were detected in various diseases, a proportion of them associated with thrombotic disorders [6,8,14-18]. In a particular patient with a past history of mesenteric vein thrombosis and moderate titers of anti-phospholipid antibodies referring for acute myocardial infarction, we hypothesized that APL could have prompted chronic cell stimulation and persisting MP shedding, leading to enhanced thrombin generation possibly accounting for unusual extensive intracardiac thrombi. Circulating levels of procoagulant MP were measured and their cellular origin determined at distance of the acute event. Values were compared to those of comparable patients presenting myocardial infarction but no history of APS.

## Case report patients treatments and methods

The patient was a 42-year-old man presenting acute myocardial infarction (STEMI). The year before, he had developed mesenteric ischemia due to mesenteric vein thrombosis and anticoagulant anti-phospholipid antibodies were evidenced. He was treated by sigmoïdectomy. No treatment by vitamin K antagonist was initiated following the former episode of mesenteric vein thrombosis.

Nine patients with STEMI and no past history of APS constituted a reference group. In this control subset, the absence of circulating APL (<10 GPL/ml) was verified on one single occasion.

All patients received the same treatment namely, percutaneous transluminal coronary angioplasty (PTCA), stent implantation, anti-platelet and anti-thrombin drugs (clopidogrel, aspirin, abciximab and low molecular weight or unfractionned heparin). Fifty healthy volunteers (HV) were simultaneously investigated. Investigations were approved by the local Ethic Committee.

## Isolation of circulating MP and determination of their procoagulant potential

Blood samples were collected on 12.9 mM tri-sodium citrate. Platelet-poor plasma (PPP) samples containing circulating MP were obtained by double centrifugation as previously described [17-19]. Procoagulant MP were captured onto insolubilized annexin V and their PhtdSer content was measured by functional prothrombinase assay using a microplate reader equipped with kinetics software. In this assay, blood clotting factor (FXa, FVa, FII) and calcium concentrations were determined to ensure that PhtdSer is the rate-limiting parameter in the generation of soluble thrombin from prothrombin. FVa was in excess with respect to FXa in order to exclude any contribution of FVa, possibly associated with MP. Results were expressed as PhtdSer equivalent (PhtdSer Eq.) by reference to a standard curve constructed with liposomes of known PhtdSer concentrations. This purified system does not allow the capture of lipoproteins, and the eventual presence of TF on captured MP does not alter values corresponding to PhtdSer content, as it is based on a true prothrombinase assay [19].

## Search for the cellular origin of circulating MP

Biotinylated monoclonal antibodies (anti-CD31 mainly for endothelial cells, anti-GPIb for platelets), were insolubilized onto streptavidin-coated microtitration plates and incubated with PPP. After washing, captured procoagulant MP were quantified by prothrombinase assay as described above. Background values obtained with irrelevant IgGs of corresponding isotype were substracted [19]. CD31 being also expressed to small extent on platelets, it was previously ensured that circulating MP bearing CD31 mainly originate from endothelial cells and are therefore a reliable marker of endothelial damage [16]. No direct comparison between capture by annexin V and antibodies can be afforded because affinities for the respective ligands are different. It has to be indicated that truly soluble forms of membrane antigens do not generate prothrombinase activity. The method enables specific measurement of procoagulant phospholipids attached to each MP phenotype.

## Characterization of anti-phospholipid antibodies

The presence of anti-phospholipid antibodies was established according to the recommendations of the Standardization SubCommittee of the International Society for Thrombosis and Haemostasis using diluted plasma coagulation assays using a 3 step procedure: (i) diluted plasma coagulation assays : dilution of patient plasma into normal plasma (Rossner index), (ii) diluted thromboplastin time, (iii) diluted Russell's Viper Venom Time (DRVVT). Briefly, the presence of circulating anticoagulant antibodies was evidenced by the LAC screen test and the presence of anti- phospholipids was confirmed in a neutralizing assay (LAC confirm) as recommended by the manufacturer (Instrumentation Laboratories, Lexington, USA). A DRVVT normalized ratio [(DRVVT screen sample/DRVVT screen normal plasma)/(DRVVT confirm sample/DRVVT confirm normal plasma)] > 1.2 was considered positive, indicating the presence of lupus anticoagulant.

Antiphospholipid immunoglobulins (IgG, IgM) were measured by enzyme immunosorbent assay as recommended by the manufacturer (Pasteur Diagnostics, Paris, France). The presence of APL was verified on two separate occasions one year apart.

## Results

**Figure 1:**
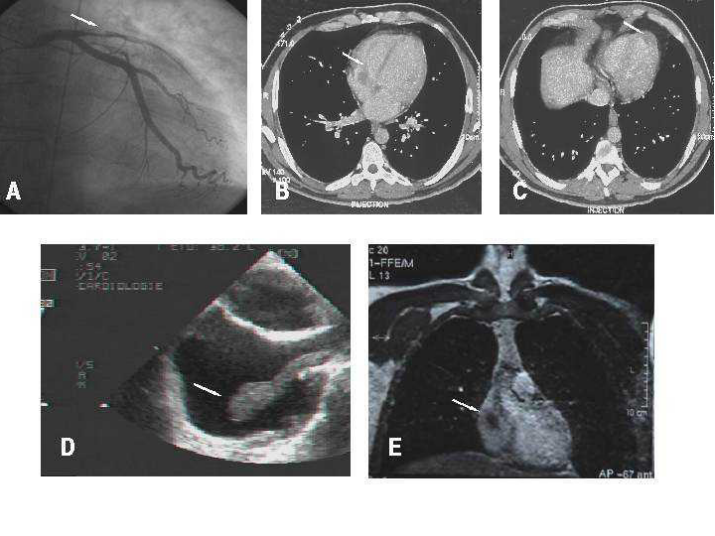
At patient admission, left coronary angiogram revealed thrombotic obstruction of left anterior descending artery (A). Large thrombus in the right auricle emerging through tricuspid valve within right ventricle (B) and apical thrombus complicating anterior aneurysm (C) detected by computer tomography scan, performed the next day. Large thrombus emerging from superiour vena cava and prolabing in the right auricle, evidenced by Trans-oesophagial echocardiography (D). Extensive thrombus emerging from superiour vena cava within right auricle, evidenced by magnetic resonance imaging (E).

The patient, with a previous history of mesenteric vein thrombosis (see methods), was admitted to our hospital with a six hours anterior myocardial infarction (STEMI). Angio-coronarography revealed left anterior descending coronary artery (LAD) and diagonal branch thrombotic occlusion (Figure 1A). The right coronary and circumflex arteries were devoid of any atherosclerotic lesion. PTCA and stent placement were performed under abciximab treatment. The issue was satisfactory (TIMI flow grade 3). Anti-thrombin and -platelet treatments were started immediately, oral anticoagulation by vitamin-K antagonists was initiated at day five. Hyper-leukocytosis (15,500/mm^3^) and a normal platelet count (236,000/mm^3^) were observed together with a creatine kinase peak at 6267 IU/l and troponin I elevation (27 ng/ml). Anticardiolipin IgG and IgM (17 and 15 GPL/ml, respectively; 16.5 and 12 GPL/ml one year ago, normal value < 10 GPL/ml) were present. Anticoagulant antibodies could be evidenced (DRVVT normalized ratio = 1.4, see methods). No anti beta-2 glycoprotein I or anti-PF4 (platelet factor 4) immunoglobulin could be detected. No systemic lupus erythematosus could be evidenced (antinuclear antibody titer below 1/80, absence of anti-DNA antibody). Coagulation parameters (antithrombin, protein C, protein S) were within the physiological range. Facteur V Leiden was excluded.

Trans-thoracic echocardiography (TTE) and computed tomography (CT) scan performed the next day revealed a complicated large anterior aneurysm with apical thrombus and a right auricle and ventricle thrombus localization. Severe left ventricular dysfunction was evidenced by a reduced ejection fraction (0.35) (Figures 1B,C). Trans-esophageal echo-cardiography and magnetic resonance imaging (MRI) confirmed the presence of large thrombi emerging from the superior vena cava and prolapsed within the right auricle (Figure 1D,E). No pulmonary embolism could be initially evidenced. One month later, the absence of intra-cardiac thrombus was verified by TTE. A distal pulmonary embolism was however revealed by helical CT scan.

## Measurement of procoagulant microparticles (MP)

In the patient, high amounts of procoagulant MP were still detectable by capture onto annexin V at day 20, at distance of STEMI. MP levels showed a two-fold elevation with respect to values observed 5 days after STEMI in a subset of comparable patients without APS. Values were 9-times higher than those measured in 50 healthy volunteers (Patient: 21 *vs*. STEMI without APS: 9.7 ± 1.5 *vs *HV: 2.3 ± 0.1 nM PhtdSer).

Procoagulant MP were mainly of platelet and endothelial origin (7.7 and 0.5 nM PhtdSer, respectively) [20,21]. MP levels appeared respectively 6- and 3-fold higher than those detected in the subset of STEMI patients with no APS. Furthermore, levels were respectively 13- and 25-fold greater than values detected in HV (Table 1). Endothelial-derived MP were highly elevated with respect to other STEMI patients, suggesting a specific contribution to thrombotic propensity in APS.

**Table 1:** Circulating microparticles (MP) in patient with APL (day 20) and in STEMI patients without APL (n = 9, day 5).

	Circulating procoagulant microparticles (nM PhtdSer Eq.)		
	
	STEMI patient with APS	STEMI patients without APL (n = 9)	Healthy Volunteers (n = 50)
MP captured onto annexin V	21	9.7 ± 1.5	2.3 ± 0.1
MP of platelet origin (captured onto anti GPIb antibody)	7.70	1.32 ± 0.18	0.58 ± 0.1
MP of endothelial origin (captured onto anti CD31 antibody)	0.50	0.17 ± 0.05	0.02 ± 0.006

Microparticles were captured onto insolubilized annexin V or on anti GPIb and anti CD31 antibodies to identify the respective contribution of platelet and endothelial-derived MP. MP catalytic phospholipid content was measured by prothombinase assay as nM Phosphatidylserine equivalents (PhtdSer Eq.). All STEMI patients were treated by PTCA, under abciximab coverage. No direct comparison between levels of MP captured onto annexin V and antibodies can be performed because affinities for the respective ligands are certainly different.

## Discussion

Testifying to major vascular damage, levels of circulating MP measured in the patient are in accordance with the important platelet and endothelial activation reported in APS [6,15]. However, such values are unexpected when detected at distance of STEMI (day 20). Indeed, platelet-derived MP were evidenced by capture on fibrinogen, as a peak occurring 8 hours after PTCA and returning to baseline within 48 h [22]. Procoagulant MP revealed by capture onto annexin V, remain slightly detectable 8 days after STEMI [16]. The persisting and combined effects of endothelial and platelet activation evidenced in the patient suggest a pivotal role in the development of unusual extensive intracardiac thrombi, despite important anti-thrombin and anti-platelet treatments.

Elevated levels of procoagulant MP are pathogenic markers of APS as well as thrombotic propensity. Thus, in this particular case, the true initiator of the intracardiac thrombi remains difficult to determine. The presence of APL was recently associated with elevated plasma levels of procoagulant MP of endothelial origin. Pointing to their possible role in thrombotic propensity, endothelial cells were shown to specifically release MP with high procoagulant activity when treated by the plasma from APS patients [15]. In our patient, moderate APL titers combined to drastic and persisting MP elevation may have impede the benefits of anti-thrombotic drugs, their decreasing expected effects on MP release [23-25] being overwhelmed by a sustained vascular stimulation [1,6,15]. Measurement of MP could be valuable in the follow up of the thrombotic propensity in patients with anti-phospholipid antibodies. The pharmacological control of MP release might constitute the next challenging issue in pathologies involving vascular cell damage and remodeling [8,9,14].

## Competing interests

The author(s) declare that they have no competing interests.
